# An Innovative Malaxer Equipped with SCADA Platform for Improving Extra Virgin Olive Oil Quality

**DOI:** 10.3390/s22062289

**Published:** 2022-03-16

**Authors:** Mariangela Vallone, Giuseppe Aiello, Filippa Bono, Claudio De Pasquale, Giovanni Presti, Pietro Catania

**Affiliations:** 1 Department of Agricultural, Food and Forest Sciences, University of Palermo, Viale Delle Scienze Ed. 4, 90128 Palermo, Italy; mariangela.vallone@unipa.it (M.V.); claudio.depasquale@unipa.it (C.D.P.); 2 Department of Engineering, University of Palermo, Viale Delle Scienze Ed. 9, 90128 Palermo, Italy; giuseppe.aiello03@unipa.it; 3 Department of Economics, Business and Statistics, University of Palermo, Viale Delle Scienze Ed. 13, 90128 Palermo, Italy; filippa.bono@unipa.it; 4 Chemical Laboratory of Palermo, Italian Customs and Monopolies Agency, Via Crispi, 143, 90133 Palermo, Italy; giovanni.presti@agenziadogane.it

**Keywords:** biophenols, control, monitoring, malaxation, oxygen, volatile compounds

## Abstract

Agriculture 4.0 is gaining more attention, and all companies are thinking about innovating machines to increase income and improve the quality of the final products. In the agro-food sector, there is space for innovation, as it is far behind the industrial sector. This paper reports an industrial-scale study on the application of an innovative system for the extraction of Sicilian EVOO (extra virgin olive oil) to improve both process management and the quality of the product. Based on previous studies, the authors suggested an innovative machine equipped with a SCADA (supervisory control and data acquisition system) for oxygen and process duration monitoring and control. The objective of the research was thus to discuss the development of a SCADA platform applied to the malaxer and the establishment of an optimized approach to control the main process parameters for obtaining high-quality EVOO. The SCADA system application in the EVOO extraction process allowed a qualitative improvement of the Sicilian EVOO of Nocellara del Belice and Cerasuola cultivars. The use of the innovative system made it possible to increase the values of tocopherols (by about 25%) in Cerasuola cultivar and total phenol content (by about 30%) in Nocellara del Belice cultivar EVOOs.

## 1. Introduction

Extra virgin olive oil (EVOO) is a key element of the Mediterranean diet [1], and its consumption is increasing worldwide because of its high content of phenolic and volatile compounds with health-promoting and sensory properties [2,3,4]. 

EVOO is obtained from the fruit of the olive tree (*Olea europaea* L.) solely by mechanical processes [5]. Among these, malaxation is the crucial step that affects the quality of the product, as numerous studies in recent years have proven [6,7,8,9,10]. During malaxation, the paste is slowly mixed at mild temperatures (generally 25–40 °C) for several minutes (30–45 min). The above conditions allow the oil droplets to come into contact with one another and coalesce. At the same time, chemical compounds are transferred between the solid, aqueous, and oil phases. Moreover, chemical and enzymatic reactions such as oxidation and hydrolysis take place affecting the olive oil chemical, nutritional, and sensory characteristics as well as the oil extraction yield [5]. A recent study [11], performed at a laboratory scale to analyze the effects of different temperatures (20, 25, and 30 °C) and times (30 and 45 min) of malaxation on EVOO phenol content, demonstrated that the highest quality and highest content of phenolic compounds were obtained by 30 min of malaxation at 20 °C; an increasing trend of oxidation with temperature and time was also found. In [12], malaxation was carried out at a low temperature (18 °C) determining an overall reduction in the phenolic fraction of EVOOs while the impact on the volatile fraction, which influences the EVOO flavor, was found to be strongly cultivar-dependent. Malaxation, therefore, can enormously influence the characteristics of the final product on the basis of the process parameter management, extensively studied in the literature, i.e., time [13], temperature [14], and composition of the atmosphere in the headspace of the malaxer [15] with particular reference to oxygen. The automatic control of the three process parameters—temperature, time, and oxygen concentration—is essential for the rationalization of the EVOO extraction process.

Monitoring systems can help the agro-food sector to have products of better quality, and today, this is a priority.

The term “digital farming” is spreading more and describes the evolution of agricultural production from “precision farming” to agricultural production systems based on modern knowledge, using smart networks and big data management tools [16]. The supervisory control and data acquisition system (SCADA) for industrial plants has been used since the 1980s, and the development concerning these systems has followed hardware technological innovation [17], providing a self-governing system, based on the closed-loop concept, which is used to provide interactions among the devices involved [18].

To date, with the spread of remote sensing and Internet of Things (IoT) technologies, process monitoring and control systems are becoming a substantial element of innovation with a great application potential in the agriculture and food industry [19,20]. The implementation of new tools based on SCADA systems in agriculture was proposed in the field of irrigation. In [21], the authors realized a SCADA platform for the regulated deficit irrigation management of almond trees, while in [22], Berrú-Ayala et al. (2020) proposed a system for banana crop irrigation control. In [23], the authors proposed a SCADA system for real-time water quality monitoring with regards to physical parameters such as temperature, turbidity, and color, obtaining manpower reduction and efficiency increase in water distribution and monitoring.

In the last years, the researchers paid particular attention to the influence of oxygen availability during malaxation on the healthy and organoleptic quality of EVOOs. The new machines introduced into the market work in sealed conditions in order to control or reduce oxygen concentration to inhibit the activities of endogenous enzymes such as polyphenoloxidase (PPO) and peroxidase (POD), which are responsible for the enzymatic degradation of phenolic compounds. In [6], the role played by the malaxation atmosphere in EVOO shelf life as a function of the storage atmosphere was studied; the authors carried out malaxation (through a micro-plant with a working capacity of 25–35 kg of olives per hour) using air, nitrogen, or argon without monitoring and controlling the atmosphere composition inside the machine during olive processing (cultivar Moraiolo). Other authors [24] tested four O_2_ levels (21%, 10%, 5%, and 2% for 35 min) inside a laboratory-scale stainless-steel malaxer processing 4 kg of olives for each replication, applying a commercial multi-component gas mixing system. The headspace oxygen concentration was discontinuously measured and recorded every minute using a commercial oxygen analyzer; the influence of high-power ultrasound treatment before malaxation was also evaluated. The results show that an O_2_ level of 5% in the malaxation headspace was the best balance between olive oil extraction yield and quality for the cultivar Arbequina. These studies applied either different atmospheres inside the malaxer without the possibility of regulating the gaseous composition or discontinuous oxygen control systems. In any case, the applications carried out so far have been performed on laboratory-scale plants or mini-plants. There is a lack of industrial-scale applications of monitoring and controlling the malaxer headspace with a SCADA system in the literature.

In previous studies, the authors of the present paper examined the role of oxygen in the headspace of the malaxer [25] and developed a software, named OCM (Oxygen Control and Monitoring), allowing the acquisition and recording of the oxygen concentration in the headspace of the malaxation machine [26]. The results obtained confirm that oxygen concentration in the malaxation process plays a fundamental role, and its monitoring and control through the OCM software improved EVOO quality.

Based on the above studies, the authors suggested an innovative machine equipped with a SCADA system not only for oxygen monitoring and control but also for temperature and duration of the process monitoring and control. The objective of the research was thus to discuss the application of a SCADA platform to the malaxer and the establishment of an optimized approach to control the main process parameters for obtaining high-quality EVOOs.

## 2. Materials and Methods

### 2.1. Olives and Oil Mill Plant

The study was performed on two typical Sicilian olive cultivars named “Cerasuola” and “Nocellara del Belice” in the crop season 2019. 

**Figure 1:**
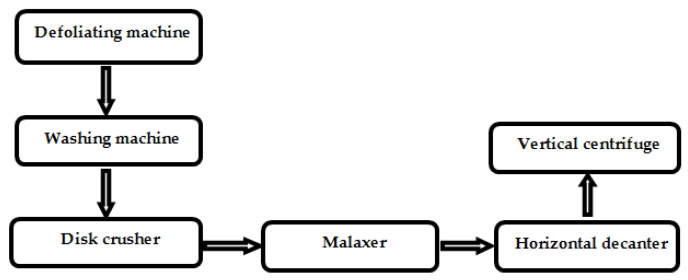
Block diagram of the olive oil mill plant.

Olives were manually harvested and processed within 24 h from harvesting in an industrial scale olive oil mill plant operating in continuous mode (Vitone Eco srl, Bitonto, Italy). The flowchart of the olive oil mill plant is shown in Figure 1.

Malaxation was carried out by adopting the process parameters recognized as optimal by numerous authors: 45 min [27] at a temperature of 27 °C [28,29,30].

The malaxer capacity was 650 L, with a 15% headspace on the total volume of the stainless steel tank. The contact surface between olive paste and air was 0.5 m^2^. The machine was equipped with the SCADA platform described in the following Section 2.2. The oil yield was about 20% in all the tests.

### 2.2. SCADA Platform

In order to monitor and control the oxygen concentration level in the headspace of the malaxation machine, a suitable industrial control system (ICS) was developed and implemented. The system is designed according to a traditional hierarchical architecture proposed by the ANSI/ISA (2010) standard [31], involving five abstraction layers from field level to enterprise management (ERP). In particular, the system proposed aims at providing a responsive solution for supervisory process control through a direct connection to actuators and sensors at the physical layer with the aim of translating the scientific knowledge about the physical and chemical transformations required to obtain a product with specified characteristics into optimized operating procedures. 

The system is thus designed to perform a periodic sampling of the atmosphere inside the malaxation machine and to return a reliable measurement of the oxygen concentration in the sample. The ICS is designed to enable multicast transmissions across different physical media and wired/wireless devices and to provide seamless integration between the programmable logic controller (PLC) and the supervisory control and data acquisition (SCADA).

**Figure 2:**
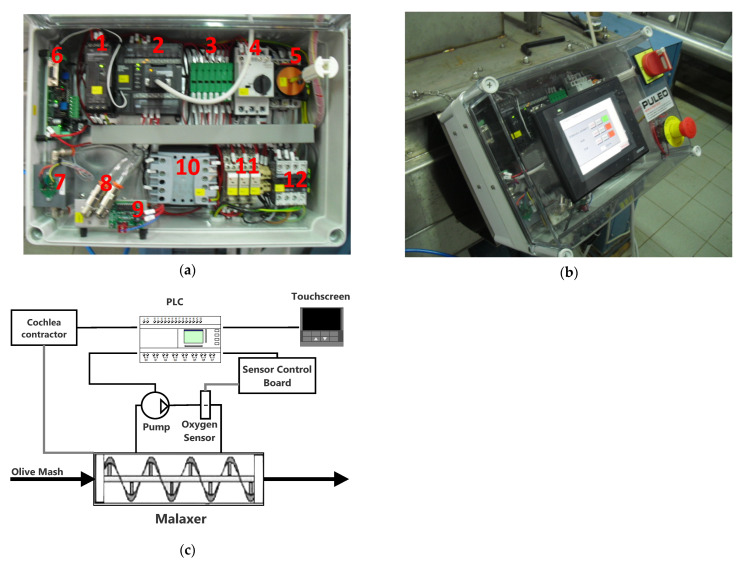
(**a**) SCADA platform inside: 1: 24 V DC power supply for PLC power supply, 2: OMRON CP1L14 PLC with relay outputs, 3: PLC protection fuses, touch panel, sampling pump, and oxygen detection board, 4: thermal-magnetic circuit breaker of the cochlea, 5: 32 A general safety switch, 6: oxygen % detection board with 0–10 V analog output (0.1–25%), 7: zirconium oxide sensor protective case, 8: Rilsan pipes for gas suction/discharge to be analyzed, 9: pump for gas sampling (flow 1 L/min), 10: 63 VA transformer, 11: activation relay: oxygen/nitrogen valves, cochlea, 12: cochlea contactor, OMRON 5.7” color touchscreen control panel; (**b**) human–machine interface; (**c**) functional block diagram.

The measurement cycle is demanded to the main control loop of the PLC (depicted in Figure 2), which extracts a gas sample from the headspace of the malaxation machine by means of a micropump and conveys it through a closed-loop pipe to the oxygen sensor.

**Figure 3:**
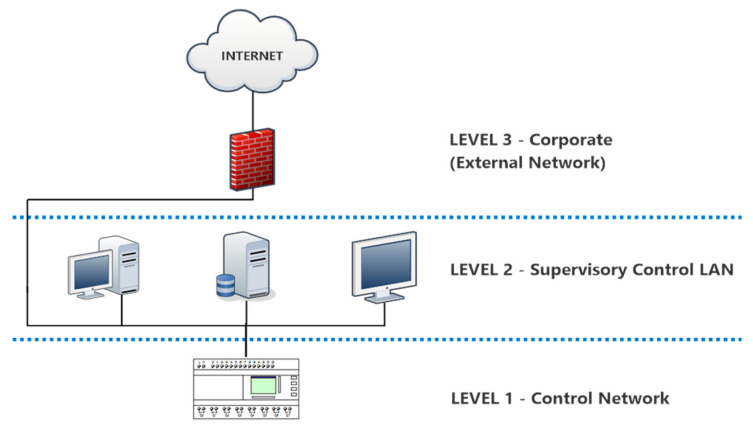
The SCADA system.

Referring to the scheme of the SCADA system (Figure 3), the oxygen monitoring circuit thus consists of a pipeline, a gas pump (1), a filter (2), and an oxygen sensor (3) [15]. The oxygen concentration detection board controls a zirconium oxide sensor which produces a 0–10 V analog output proportional to 0.1–25% physical concentration of oxygen, with an accuracy of 0.5% O_2_ after calibration with standard gases. Zirconium-oxide sensors are based on the principle of a solid-state electrochemical cell constituted by a layer of yttria-stabilized zirconium oxide allowing the movement of oxygen ions thus producing an electromotive force that is used to determine the oxygen concentration. The analog voltage output produced by each sensor is subsequently processed in the measurement chain, which consists of an analog front end (AFE) to amplify the signal from the sensor, an analog-to-digital converter (ADC) to digitize the amplified signal, and a processor to validate the sensor information.

The automated scanning system consists of a periodic sequence of operations repeatedly executed by the PLC. The cycle starts with the activation of the relay controlling the gas aspiration pump which extracts a gas sample from the malaxer and delivers it to the oxygen sensor through gas sampling loop. The suction pump is activated for 15 s by the relay, while the sensor control board activates with 5 s delay after the pump starts in order to allow the gas sample to reach the sensors. The sensor board thus performs the readings in 5 s, considering the response time of the sensor is less than 4 s. The overall PLC’s scan time (i.e., the time required to go through the complete program) is thus approximately 15 s, and it is repeated after 30 s from the beginning. 

**Figure 4:**
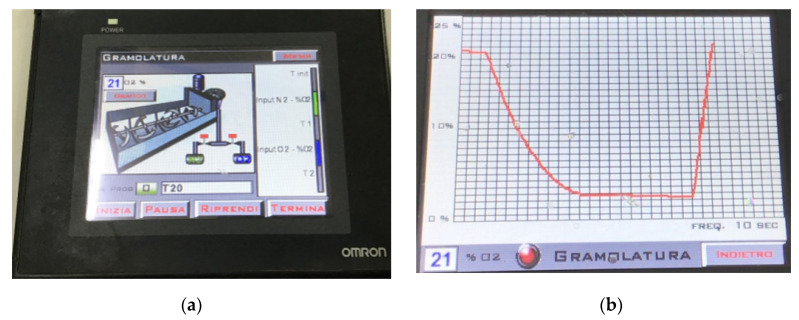
SCADA platform HMI (human–machine interface). (**a**) The HMI allows one to real-time manage the malaxing process with the automatic injection of O_2_ and N_2_ gases into the headspace of the malaxer by means of the solenoid valves present in the circuit. (**b**) The O_2_ curve in the headspace of the malaxer is monitored and controlled by the operator.

The oxygen concentration value thus obtained is pushed from the PLC to the SCADA system for data storage and processing. The dissolved oxygen measurements in the malaxation camera are performed every 30 s, and the data collected during malaxation process are displayed in real time on the standard human–machine interface (HMI) constituted by an OMRON 5.7” display (Figure 4). Clearly, considering the inertia of the gas in the sampling circuit and the related 5 s delay introduced from the pump starting event to the start of the measurement and the 5 s required for performing the concentration measure, the “real-time” value detected by the system is in fact related to the oxygen concentration in the malaxer atmosphere approximately 10 s before, which is acceptable considering the whole malaxation process has a duration of 2400 s.

The system is also connected to the Ethernet network, which allows the data gathered by the PLC to be shared via an Ethernet-IP front module (the OMRON CP1 does not have native Ethernet-ip port) with the SCADA supervisory system running on the corporate ICT infrastructure.

This Industrial EtherNet/IP network also allows for wireless communication and remote process control via portable devices (tablets, smartphones, etc.).

The measurements are thus processed by the OCM software developed by the authors [26] to acquire and record the oxygen concentration in the headspace of the malaxation machine. The control application developed is run on the Windows operating system; it allows the definition of advanced procedures to control the concentration of gases (O_2_ and N_2_) inside the malaxation machine. The percentage of oxygen values inside the malaxation headspace are shown in real time after they have been acquired by the sensors of the OCM system. The oxygen concentration inside the malaxation machine is sampled by means of a gas extraction system that continuously circulates. This was sampled through a closed-loop pipe where the oxygen sensor is located. The OCM software is provided with three screens: the first one to manage the timepoints of the process, the second one to handle the electro valves in order to insert oxygen and nitrogen inside the machine, and the third one to continuously monitor the oxygen concentration in the headspace of the malaxer.

The HMI (human–machine interface) is linked to the SCADA system’s databases and software programs to provide trending, diagnostic data and management information.

### 2.3. Experimental Trials

Olives of the cultivars Nocellara del Belice and Cerasuola were processed to perform the experimental tests. The fruit maturity was determined through the Jaen index as described in [32], obtaining a value of 1.70. The olives used in the tests had similar ripeness degree and were healthy.

Two trials were carried out, respectively named T0, unmodified malaxation, and T20, modified malaxation, with the application of the SCADA platform for both olive varieties.

The SCADA allowed modifying the atmosphere composition in the malaxation chamber headspace introducing N_2_ and/or O_2_ (pure gases) during the process. In particular, the atmosphere composition in the malaxation chamber headspace was left unmodified in test T0; in test T20, the malaxation chamber was first made inert by filling N_2_ before the olive paste entry, and then 30 L of O_2_ was introduced at 20 min from the beginning of the process.

Each test configuration was replicated three times. Olive oil samples were collected immediately after each test and stored in 0.1 L dark glass bottles at 10 °C during transport to the laboratory, where analyses were performed.

### 2.4. Analytical Determinations in Extra Virgin Olive Oils

All of the EVOO samples of the two cultivars taken from each repetition were subjected to chemical analyses. 

The main quality parameters—free acidity (FA, %), peroxide value (PV, meq O_2_/kg), UV spectrophotometric indices K232, K268, delta K (ΔK), waxes (WA ppm), total ethyl esters (TEE, ppm), total sterols (TS, ppm), total betasitosterols (TBS, %), and tocopherols (TO, ppm)—were determined according to the procedures described in the Commission Regulation (EEC) No. 2568/91.

The olive oil phenolic composition was analyzed by high-performance liquid chromatography equipped with thermostated autosampler (HPLC-MS) (Agilent, 6546 Quadrupole Time of Flight LC/MS, Santa Clara, CA, USA), and a 5 μm particle size C18 Luna column, 15 cm, 2 mm i.d. (Phenomenex, Torrance, CA, USA) was used.

Methanol, acetonitrile, and *n*-hexane were all HPLC grade purchased from Fluka; standard phenolic compounds were from Sigma Aldrich (Milano, Italy). Formic acid was from Sigma Chemical Co. (St. Louis, MO, USA). Ultrapure water was made by using a MilliQ system (Millipore, Bedford, MA, USA). MilliQ water/methanol (90/10 *v*/*v*) solvent solution was used for standard injections. Dark-brown glass bottles at 4 °C were used to store the samples until analysis procedures. The oil met the aforementioned standards set by the European Commission for extra-virgin quality. A total of 3 g of oil sample using an SPE diol cartridge (Vac RC 500 mg, Waters, Milford, MA, USA) was used to clean and concentrate the sample in order to obtain the polar fraction. Therefore 6 mL of *n*-hexane, 6 mL of methanol: water (80:20) solution, and 3 mL of acetonitrile were used to achieve the activation of SPE stationary phase. A total of 10 mL of *n*-hexane was used under vacuum to remove the nonpolar fraction from oil, and 8 mL of the before-mentioned methanol: water solution and 4 mL of acetonitrile were used to elute phenolic compounds. The vacuum was maintained at less than 30 kPa. A gentle N_2_ stream was used to evaporate the eluent to 2 mL. To filter the samples, 13 mm PTFE 0.45 um membrane filter, purchased from Waters, was used. A total of 20 μL was injected into the liquid chromatography. Brown-glass material was used during the process in order to minimize possible oxidation process.

The HPLC column was kept at ambient temperature. A binary solvent solution using water acidified with 0.1% formic acid (solvent A) and 100% acetonitrile (solvent B), kept at a flow rate of 0.5 mL min^−1^, was used as the mobile phase with a gradient program started with 90% eluent A and 10% eluent B, which ramped linearly to 25% in 12 min. This percentage value was maintained for 7 min; afterward, eluent B was ramped again linearly to 40% at 30 min and to 60% at 40 min. The linearity of the calibration method and the standard curve of each phenolic compound, fortified by triplicate analyses, were used to express the concentration ranges of the considered samples. The phenolic compounds evaluated semiquantitatively by HPLC were hydroxytyrosol (HT), tyrosol (TY), p-coumaric acid (pCA), ferulic acid (FA), tyrosol acetate (TY), pinoresinol (PN), luteolin (LU), apigenin (AP), and methyl luteolin (mLU). The total concentration of the ligostride derivatives (Ligst. Der) and the total concentration of the oleuropein derivatives (Oleur Der.) were also determined. Determination of total phenol content (TPC) was performed following Baiano et al. (2009) [33]. A total of 2 mL of methanol/water (70:30, *v*/*v*) and 2 mL of hexane were added to 5 g of oil samples and mixed with a vortex (10 min). The hydro-alcoholic phase containing phenols was separated from the oil phase by several centrifugations; 100 µL of phenolic extract was mixed with 100 µL of Folin–Ciocalteu reagent (2N) and, after 4 min, with 800 µL of an aqueous solution of Na_2_CO_3_ (5%). The mixture was heated in a 40 °C water bath for 20 min, and the total phenol content was determined calorimetrically at 750 nm. The total phenolic content was expressed as milligrams of gallic acid equivalents per kilogram of oil. These components give the greatest oxidation resistance to the oils and are influenced by the presence of oxygen during the extraction process.

Olive oil (100 mL) fatty acids were determined by a methylation reaction using 2 mL of 0.5 M sodium methoxide solution in methanol at 30 °C for 15 min and 1 mL of 5% hydrochloric acid/methanol solution at 50 °C for 15 min. *n-*Hexane (1.5 mL) was finally used to recover fatty acid methyl esters (FAME). An autosampler and an HP 6890 gas chromatography system equipped with a flame-ionization detector (FID) (Agilent Technologies Inc., Santa Clara, CA, USA) were used for the analysis procedures by the injection of 1 μL of each sample. FAME were determined using a 100 m length, 0.25 mm i.d., 0.25 μm capillary column (CP-Sil 88; Chrompack, Middelburg, The Netherlands). The injector port and the FID temperatures were kept at 255 °C and 250 °C, respectively, with a H_2_ flow of 40 mL min^−1^, air flow of 400 mL min^−1^, and a He constant flow of 45 mL min^−1^. The oven temperature ramps were 70 °C for 1 min, increased at 5 °C min^−1^ to 100 °C, held for 2 min, increased at 10 °C min^−1^ to 175 °C, held for 40 min, and finally increased at 5 °C min^−1^ to a final temperature of 225 °C and held for 45 min. A head pressure of 158.6 kPa with flow rate of 0.7 mL min^−1^ of He (linear velocity of 14 cm s^−1^) was used as carrier gas to reach the proper separation. A standard mix solution in *n*-hexane of target fatty acid methyl ester compounds was used to identify quantitatively each FA. Moreover, C23:0 (4 mg g^−1^ of oil) (Sigma Aldrich) was added to each sample as internal standard in order to quantify FA. Myristic acid (C14:0), palmitic acid (C16:0), palmitoleic acid (C16:1), margaric acid (C17:0), eptadecenoid acid (C17:1), stearic acid (C18:0), oleic acid (C18:1), linoleic acid (C18:2), arachid acid (C20:0), eicosenoic acid (C20:1), linolenic acid (C18:3), behenic acid (C22:0), lignoceric acid (C24:0), saturated fatty acids (SFA), monounsaturated fatty acids (MUFA), and polyunsaturated fatty acids (PUFA) were determined.

HS-SPME and GC–MS were used to isolate and identify the volatile fraction of olive oil samples. The target compounds were identified using an HP 5890 GC–MS equipped with the mass selective detector HP 5973. A capillary column, HP5-MS, 5% diphenyl-95% dimethylpolysiloxane (30 m 0.2 mm, 0.25 mm film thickness), was used as stationary phase. A splitless injection method with He as carrier gas at 10^−3^ L min^−1^ was used in order to reach a separation and the identification and quantification of target compounds. The injector port was at 250 °C. The oven ramp temperatures were an isotherm at 60 °C for 8 min followed by a linear increase of 4 °C min^−1^ up to 180 °C held for 2 min. MS scan conditions were a source temperature at 230 °C, an interface temperature at 280 °C, the E energy at 70 eV, and the full-scan mass range between 39 and 350 amu.

A standard mix solution of *n*-alkanes (C6–C22), obtained from FLUKA, run under the chromatographic condition described above, was used to calculate the linear retention indices. Standard mixtures of selected essential oils were injected into the GC inlet and retention indices determined. Monoterpenes, sesquiterpenes, alcohols, aldehydes, and ester of reference compounds from different classes were used to determine the response factors; it was found to range from 0.85 to 1.2 versus *n*-hexanol, averaging 1.0. Response factors were therefore taken as 1.0 for all compounds.

Singular-component standard addition experiments were carried out to evaluate matrix interference. A commercial library (NIST 2005) and an FFC (flavor and fragrance compounds) bank, provided with linear retention indices on the same column, interactively with the experimental MS data, were used to perform the oil components identification. The total content of the single compounds was calculated as percent of total chromatographic area, and the report values were the average of triplicate experiments [34]. 2,4 hexadienal, (E,E), 2-pentenal (E), hexenal, pentenal 2-(E), 1-penten-3-ol, 2-hexen-1-ol, 1-hexanol, 3-hexen-1-ol (Z) were determined. These compounds originate during the mechanical extraction process of EVOO from the lipoxygenase pathway [27].

### 2.5. Statistical Analysis

Chemical analyses of EVOO were performed on three EVOO samples for each case of studies within one week from extraction. The means and standard deviations of the experimental results were calculated from three replicate trials in the Nocellara del Belice and Cerasuola EVOOs. To determine differences in all the parameters listed in Section 2.4, for the modified (T0) and unmodified malaxation (T1) for each cultivar, t-test was used. Differences at *p* < 0.05 were considered significant and were indicated with an asterisk. Principal component analysis (PCA) was used to find the principal components contributing to most of the variance in the analytical determinations in all EVOO samples for the two cultivars. The principal components were considered significant if their eigenvalues were >1 [35] (Cattell, 1966). All statistical analyses were carried out with Stata 16.1 statistic software (StataCorp LLC, College Station, TX, USA).

## 3. Results and Discussion

The malaxer equipped with the SCADA platform was used for the production of EVOO of the Sicilian olive cultivars “Cerasuola” and “Nocellara del Belice”.

Table 1 shows the values of the main quality parameters of the EVOOs of the two cultivars: free acidity (FA, %), peroxide value (PV, meq O_2_/kg), UV K232, UV K268, delta K (ΔK), waxes (WA ppm), total ethyl esters (TEE, ppm), total sterols (TS, ppm), total betasitosterols (TBS, %), and tocopherols (TO, ppm). Statistically significant differences were found between tests T0 and T20 for the parameters FA (Cerasuola), TEE (Nocellara del Belice), and TO (Cerasuola).

Acidity is a very important parameter that gives information on the quality of processed olives.

To be defined as extra virgin olive oil, it must have an FA value of less than 0.8%, but high-quality EVOOs must have values not exceeding 0.1–0.2%. Higher acidity values are attributed to olive fruit fly attacks, poor conservation, and the presence of other defects on the fruit. The FA values of the EVOOs were higher in the Cerasuola cultivar than in Nocellara del Belice. The FA value obtained in test T20 for the Cerasuola cultivar is very interesting; the SCADA platform, in fact, allowed lowering the FA value of this test by about 6% compared to the value recorded in test T0. This is a very important topic since, in the literature to date, there are no studies with similar results. It could be justified by the targeted presence of oxygen in the headspace of the malaxer that would have limited the action of the lipase enzyme responsible for the hydrolysis of triglycerides [36].

Another important parameter that allows one to define the quality of EVOO is total ethyl esters (TEE). The concentration of this parameter in EVOO must not exceed 35 mg/kg. It represents an important factor since it can be directly related to the contents of free fatty acids and ethanol in EVOO. The higher the TEE value, the lower the quality of the EVOO; this is because ethanol is a substrate for the chemical synthesis of ethyl esters of fatty acids [37]. The TEE value obtained in test T20 of the Nocellara del Belice cultivar was 75% lower than that obtained in test T0.

The nutritional value of EVOO is derived from high levels of oleic acid and from minor components such as phytosterols, carotenoids, tocopherols, and hydrophilic phenols [38]. Tocopherol takes on significant importance in the diet for both antioxidant activity and vitamin properties. Tocopherols are present in variable quantities within the fraction of EVOO minor constituents. For example, Beltrán et al. (2010) in [39] reported a total tocopherol content between 84 and 463 ppm by studying thirty cultivars of EVOO, monitored during fruit ripening for three consecutive growing seasons. The TO values recorded in test T0 of the two varieties under study were not high, 150 ppm in Nocellara del Belice and 128 ppm in Cerasuola cv. The application of the SCADA system improved the TO values in the Cerasuola cultivar with an increase of about 25% compared to test T0.

The PCA carried out among the main quality parameters of the EVOOs highlights two main components that together explain 94% of the overall variability of EVOOs (Table 2).

**Figure 5:**
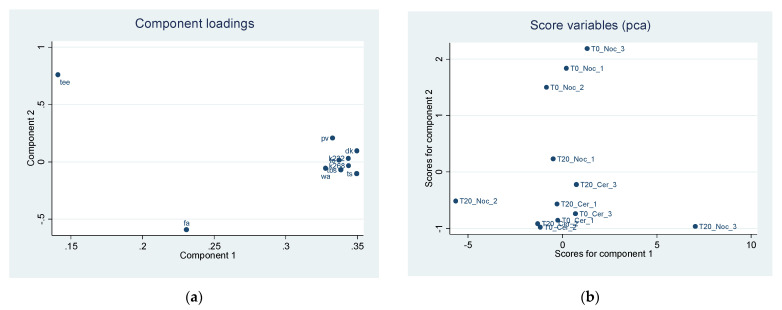
(**a**) Component loadings of quality parameters in two EVOO cultivars by different malaxation methods (T0 and T20). First two principal component (Component 1 *x*-axis, Component 2 *y*-axis) analysis (PCA). (**b**) Predicted scores after PCA obtained as linear combination of the observation data by using eigenvector of component 1 (*x*-axis) and eigenvector of component 2 (*y*-axis).

The first component is determined in the same proportion by the elements PV, K232, K268, ΔK, WA, TS, TBS, and TO. The second component has a positive correlation with TEE and a negative with FA. Component loadings, explaining the position of each quality parameter in the first two components, are reported in Figure 5a. Table 3 shows all the component loadings of the quality parameters of EVOOs.

The scoreplot (Figure 5b), reporting the estimated scores employing the first two principal components extracted after PCA, highlights a clear separation of the EVOO characteristics of the two tests on component 2, while no difference seems to exist on component 1. In particular, test T20 of the Nocellara del Belice cultivar shows lower values of the second component compared to test T0. This shows the higher-quality characteristics of Nocellara EVOO obtained with the innovative malaxation system, while there seems to be no difference in the Cerasuola cultivar for either of the two components.

As regards the biophenols, Table 4 shows the values of the main components recorded in the two tests, T0 and T20, for the two varieties.

Given the not very high quality conditions of the 2019 olive crop season, the SCADA system applied to the malaxer, in particular, could improve the content of biophenols in the Nocellara del Belice EVOO. The compounds that showed significantly higher values in T20 compared to T0 were HT, pCA, FA, Ligst. Der., and TPC. HT was even doubled; in fact, 9.78 mg/kg was obtained in T20 while 4.65 mg/kg in T0. HT is one of the best phytochemical compounds known so far with high-antioxidant properties and the only phytochemical with EFSA (European Food Safety Authority)-approved health benefits and safety [40].

Overall, in the Nocellara del Belice cultivar, thanks to the application of SCADA, the TPC values in T20 increased by about 43% compared to T0. It is important to emphasize that the phenolic compounds of EVOO include preventive chemo activity [41]. The anticarcinogenic activity of biophenols may be due not only to their antioxidant properties but also to their ability to reduce the bioavailability of carcinogens and inhibit their metabolic activation [42,43]. Moreover, the biophenol increase in Nocellara del Belice EVOO could improve its shelf life because it avoids lipid oxidation through several mechanisms based on radical scavenging, hydrogen atom transfer, and metal chelation [44].

The analysis of the main components carried out on the biophenols highlights two main components that together explain 91% of the overall variability of EVOOs (Table 5). The first component has a high and positive correlation with TYac, mLU, and Oleur Der. and negative with TY, whereas the second component correlates with FA, TPC, and HT.

**Figure 6:**
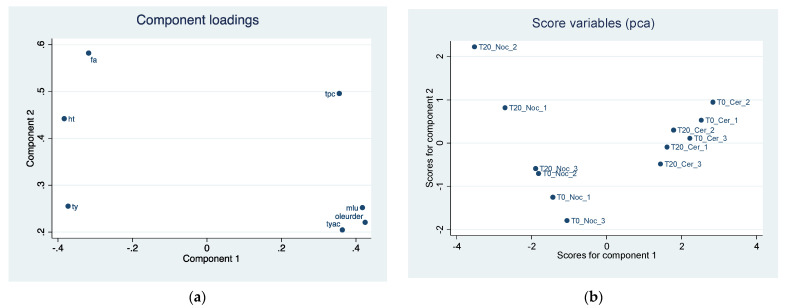
(**a**) Component loadings of biophenols in two EVOO cultivars by different malaxation methods (T0 and T20). First two principal component (Component 1 *x*-axis, Component 2 *y*-axis) analysis (PCA). (**b**) Predicted scores after PCA obtained as linear combination of the observation data by using eigenvector of component 1 (*x*-axis) and eigenvector of component 2 (*y*-axis).

Component loadings, explaining the position of biophenols in the first two components, are reported in Figure 6a. The scoreplot (Figure 6b) highlights a clear separation of the characteristics of the EVOOs on the two components. Nocellara del Belice EVOO obtained according to T20 shows higher values of component 2 than T0. The Cerasuola EVOO extracted according to T20 has lower values of component 2 compared to T0; the level of component 1 in the two tests is not very different in both Nocellara del Belice and Cerasuola.

Table 6 shows all the component loadings of the biophenols of EVOOs.

Table 7 shows the fatty acid profile of the EVOOs under consideration.

Monounsaturated fatty acids (MUFA) have been proven to reduce LDL (low-density lipoprotein) cholesterol, while they may increase HDL (high-density lipoprotein) cholesterol (FAO/WHO 2010) [45]. Oleic acid (C18: 1, n-9) can promote insulin resistance in contrast to polyunsaturated fatty acids (PUFA) with protection against insulin resistance (FAO/WHO 2010) [45]. Overall, the application of the SCADA platform did not affect the fatty acid profile of the Nocellara del Belice and Cerasuola EVOOs obtained.

Principal component analysis among the fatty acids shows two principal components together explaining 93% of the overall variability of EVOOs (Table 8). In the first component, myristic acid, margaric acid, eptadecenoid acid, linolenic acid, arachid acid, and lignoceric acid contribute to determining it in equal measure. The second component has a positive correlation with oleic acid, eicosenoic acid, and arachid acid and a negative correlation with palmitic and linoleic acids.

**Figure 7:**
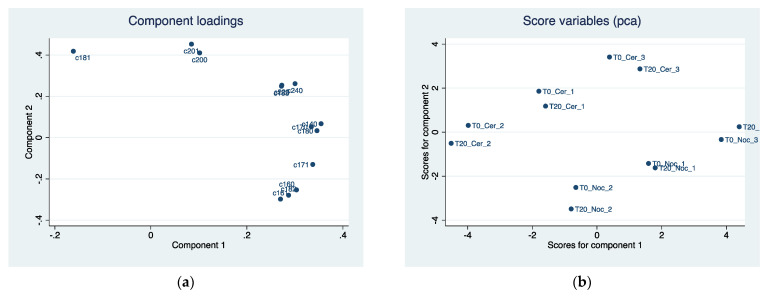
(**a**) Component loadings of fatty acid components in two EVOO cultivars by different malaxation methods (T0 and T20). First two principal component (Component 1 *x*-axis, Component 2 *y*-axis) analysis (PCA). (**b**) Predicted scores after PCA obtained as linear combination of the observation data by using eigenvector of component 1 (*x*-axis) and eigenvector of component 2 (*y*-axis).

Component loadings, explaining the position of each fatty acid in the first two components, are reported in Figure 7a. The scoreplot in Figure 7b highlights a clear separation of the characteristics of the two oils on component 2, while no difference seems to exist on component 1. Test T20 recorded lower values of component 2 for both varieties. Table 9 shows all the component loadings of the fatty acids of EVOOs.

Table 10 shows the amount of volatile compounds of Nocellara del Belice and Cerasuola EVOOs. C6 volatile compounds (aldehydes and alcohols) that originated in the lipoxygenase pathway are connected to positive sensory characteristics of olive oil and contribute to the green olive oil aroma [46]. C5 volatile compounds, which originate in the additional branch of the lipoxygenase pathway, contribute to the pleasant aroma and positively correlate with the bitterness and pungency of EVOO [46].

Tests T20 showed C6 and C5 compound values higher than those obtained in tests T0 for both varieties. A 55% and 41% increase is noted for the Nocellara del Belice cultivar, respectively, for the compounds 2,4 hexadienal, (E,E) and 1-penten-3-ol. For the Cerasuola cultivar, there was an increase in 2,4 hexadienal, (E,E) (+40%), 9, hexanal (+21%), 1-penten-3-ol (+38%), 2-hexen-1-ol (+45%), and 1-hexanol (+13%). The SCADA platform applied to the malaxer allowed increasing C5 and C6 volatile compounds (aldehydes and alcohols) for both varieties. The C5 and C6 volatile compound higher values greatly improved the quality of EVOOs in terms of sensory notes (cut grass, artichoke, tomato, rosemary, etc.). For the Sicilian EVOO varieties that generally have a low quantity of such compounds, the SCADA system application plays an important role to make the EVOO more balanced in terms of polyphenols and volatile compounds.

Principal component analysis among volatile compounds shows two principal components together explaining 98% of the overall variability of EVOOs (Table 11).

The first component is determined in the same proportion by the elements hexanal, pentenal 2-(E), hexanal, 3-hexen-1-ol (Z). The second component positively correlates with 2,4 hexadienal, (E,E) and 2-pentenale (E).

**Figure 8:**
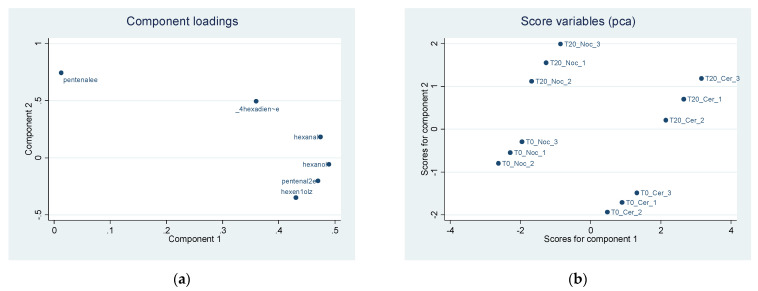
(**a**) Component loadings of volatile compounds in two EVOO cultivars by different malaxation methods (T0 and T20). First two principal component (Component 1 *x*-axis, Component 2 *y*-axis) analysis (PCA). (**b**) Predicted scores after PCA obtained as linear combination of the observation data by using eigenvector of component 1 (*x*-axis) and eigenvector of component 2 (*y*-axis).

Component loadings, explaining the position of each volatile compound in the first two components, are reported in Figure 8a. The scoreplot (Figure 8b) highlights a clear separation of the two EVOOs’ volatile compounds in both component 1 and component 2. Test T20 leads to higher values of component 2 both in Nocellara del Belice and Cerasuola cultivars. In component 1, the highest values for T20 concern only Cerasuola, while they are unchanged for the Nocellara del Belice cultivar.

Table 12 shows all the component loadings of the volatile compounds of EVOOs.

## 4. Conclusions

The SCADA system application in the EVOO extraction process produced a qualitative improvement of the Sicilian EVOOs of Nocellara del Belice and Cerasuola cultivars. The system made it possible to increase the values of tocopherols (by about 25%) in the Cerasuola cultivar and total phenol content (by about 30%) in the Nocellara del Belice cultivar. This result is extremely important since the Nocellara del Belice cultivar generally has a limited content of TPC compared to other Sicilian cultivars such as Cerasuola and Biancolilla. The volatile components of the EVOOs of the two varieties were clearly improved in the tests where the SCADA system was applied. In particular, higher values were recorded for all C5 and C6 volatile compounds (aldehydes and alcohols).

The study, therefore, made it possible to record a further step forward in olive oil plant engineering in order to improve the quality of Sicilian EVOOs. 

## Figures and Tables

**Table 1:** Quality parameters (mean ± SD) of Nocellara del Belice and Cerasuola EVOOs for tests T0 (unmodified malaxation) and T20 (modified malaxation, with the application of the SCADA platform).

	Nocellara del Belice				Cerasuola			
	T0		T20		T0		T20	
FA	0.29	±0.02	ns 0.26	±0.03	0.56	±0.01	0.47 *	±0.02
PV	10.0	±2.0	8.0	±2.0	8.0	±1.0	6.0	±2.0
K232	2.0	±0.12	1.93	±0.08	1.77	±0.12	1.91	±0.1
K268	0.18	±0.02	0.2	±0.05	0.16	±0.05	0.18	±0.01
ΔK	0.006	±0.001	0.005	±0.001	0.005	±0.001	0.005	±0.001
WA	57.0	±18.0	49.0	±21.0	59.0	±18.0	53.0	±19.0
TEE	31.0	±5.0	7.75 *	±2.0	7.4	±2.0	8.2	±5.0
TS	1227.0	±194.0	1277.0	±125.0	1308.0	±142.0	1258.0	±186.0
TBS	94.76	±1.5	94.83	±0.8	94.82	±0.92	94.82	±0.5
TO	150.0	±18.0	144.0	±21.0	128.0	±20.0	170.0 *	±16.0

SD: Standard deviation of three replicates, FA: Free acidity (%), PV: Peroxide value (meq O_2_/kg), K232: UV, K268: UV, ΔK: delta K, WA: Waxes (ppm), TEE: Total ethyl esters (ppm), TS: Total sterols (ppm), TBS: Total betasitosterols (%), TO: Tocopherols (ppm). The asterisk indicates statistically significant differences at a significance level of 5%.

**Table 2:** Eigenvalues and variance explained by the first two principal components for the quality parameters of EVOOs.

Component	Eigenvalue	Difference	Proportion	Cumulative
1	8.042	6.670	0.804	0.804
2	1.373	1.060	0.137	0.942

**Table 3:** Component loadings of the quality parameters of EVOOs.

Parameters	Comp1	Comp2	Comp3	Comp4	Comp5	Comp6	Comp7	Unexplained
FA	0.231	−0.590	0.396	0.474	0.348	0.197	0.194	0.111
PV	0.333	0.207	0.236	0.215	−0.446	−0.530	0.357	0.209
K232	0.344	0.030	−0.374	0.174	0.031	0.053	−0.480	0.690
K268	0.344	−0.034	−0.212	−0.279	−0.318	0.636	0.489	0.091
ΔK	0.350	0.097	0.082	−0.076	−0.046	0.134	−0.299	−0.331
WA	0.328	−0.055	0.579	−0.411	−0.118	0.058	−0.417	−0.033
TEE	0.141	0.761	0.239	0.313	0.344	0.279	0.093	−0.025
TS	0.349	−0.101	−0.054	0.003	−0.095	−0.256	0.071	−0.126
TBS	0.338	−0.071	−0.405	0.372	−0.050	−0.036	−0.148	−0.577
TO	0.337	0.014	−0.192	−0.458	0.658	−0.326	0.256	0.017

**Table 4:** Concentration of the biophenols (mean ± SD) of Nocellara del Belice and Cerasuola EVOOs for tests T0 (unmodified malaxation) and T20 (modified malaxation, with the application of the SCADA platform). Data are expressed in ppm.

	Nocellara del Belice				Cerasuola			
	T0		T20		T0		T20	
HT	4.65	±1.5	9.78 *	±3.2	2.16	±0.4	1.56	±0.7
TY	6.2	±3.5	7.18	±4.1	1.11	±0.8	1.13	±0.55
pCA	0.0	±0.0	5.29 *	±1.29	0.0	±0.0	0.0	±0.0
FA	0.0	±0.0	6.18 *	±2.54	0.0	±0.0	0.0	±0.0
TY ac	2.26	±0.54	0.0	±0.0	13.39	±1.9	3.94 *	±0.4
PN	23.89	±5.5	26.22 *	±4.7	42.78	±5.3	58.69 *	±6.2
LU	0.0	±0.0	6.35 *	±1.2	8.5	±1.3	9.43	±1.1
AP	0.0	±0.0	0.0	±0.0	13.52	±1.9	5.16 *	±0.84
mLU	0.0	±0.0	0.0	±0.0	13.83	±3.1	12.19	±1.8
Ligst. Der.	47.48	±2.5	73.25 *	±3.5	73.56	±5.2	93.61 *	±5.4
Oleur Der.	22.37	±1.1	17.52 *	±1.5	92.94	±2.5	73.83 *	±3.5
TPC	106.8	±15.0	152.0 *	±16.0	260.0	±17.0	259.0	±15.0

SD: Standard deviation of three replicates, HT: Hydroxytyrosol, TY: Tyrosol, pCA: p-Coumaric acid, FA: Ferulic acid, TY: Tyrosol acetate, PN: Pinoresinol, LU: Luteolin, AP: Apigenin, mLU: Methyl luteolin, Ligst. Der.: Total concentration of the ligostride derivatives, Oleur Der.: Total concentration of the oleuropein derivatives, TPC: Total phenol content. The asterisk indicates statistically significant differences at a significance level of 5%.

**Table 5:** Eigenvalues and variance explained by the first two principal components for the biophenol extracts of EVOOs.

Component	Eigenvalue	Difference	Proportion	Cumulative
1	5.188	4.028	0.741	0.741
2	1.160	0.747	0.1696	0.907

**Table 6:** Component loadings of the biophenols of EVOOs.

Variable	Comp1	Comp2	Comp3	Comp4	Comp5	Comp6	Comp7	Unexplained
HT	−0.383	0.442	0.115	−0.113	0.235	−0.704	0.285	0
TY	−0.373	0.255	0.526	0.636	−0.092	0.325	0.022	0
FA	−0.318	0.582	−0.313	−0.437	−0.118	0.506	−0.030	0
TYac	0.363	0.205	0.726	−0.469	0.059	0.034	−0.271	0
mLU	0.417	0.252	−0.105	0.191	0.732	0.262	0.331	0
Oleur Der.	0.425	0.221	0.038	0.037	−0.599	−0.034	0.638	0
TPC	0.355	0.496	−0.269	0.366	−0.153	−0.267	−0.571	0

**Table 7:** Fatty acid profile (mean ± SD) of Nocellara del Belice and Cerasuola EVOOs for tests T0 (unmodified malaxation) and T20 (modified malaxation, with the application of the SCADA platform). Data are expressed in %.

	Nocellara del Belice				Cerasuola			
	T0		T20		T0		T20	
C14:0	0.02	±0.01	0.02	±0.01	0.01	±0.01	0.01	±0.01
C16:0	13.25	±0.745	13.17	±0.55	10.04	±0.55	10.17	±0.64
C16:1	1.18	±0.08	1.08 *	±0.05	0.47	±0.05	0.67	±0.09
C17:0	0.13	±0.02	0.14	±0.04	0.08	±0.03	0.1	±0.08
C17:1	0.25	±0.05	0.25	±0.02	0.16	±0.02	0.18	±0.05
C18:0	3.21	±0.40	3.28	±0.50	2.62	±0.40	2.94	±0.40
C18:1	71.06	±0.80	70.35	±0.55	76.78	±0,98	76.77	±1.11
C18:2	9.16	±0.21	9.55	±0.36	7.43	±0.21	7.47	±0.33
C20:1	0.28	±0.05	0.27	±0.11	0.39	±0.05	0.34	±0.09
C20:0	0.4	±0.03	0.38	±0.05	0.42	±0.06	0.46	±0.05
C18:3	0.71	±0.10	0.7	±0.12	0.74	±0.08	0.62	±0.14
C22:0	0.13	±0.02	0.14	±0.03	0.14	±0.03	0.12	±0.02
C24:0	0.11	±0.05	0.11	±0.06	0.1	±0.05	0.1	±0.06
SFA	17.25	±0.18	17.24	±0.18	13.41	±0.16	13.9	±0.18
MUFA	72.77	±0.24	71.95	±0.18	77.8	±0.27	77.96	±0.33
PUFA	9.87	±0.15	10.25	±0.24	8.17	±0.14	8.09	±0.23
C18:1/C18:2	7.76		7.37		10.33		10.28	
MUFA/PUFA	7.37		7.02		9.52		9.64	

SD: Standard deviation of three replicates, C14:0: Myristic acid, C16:0: Palmitic acid, C16:1: Palmitoleic acid, C17:0: Margaric acid, C17:1: Eptadecenoid acid, C18:0: Stearic acid, C18:1: Oleic acid, C18:2: Linoleic acid, C20:0: Arachid acid, C20:1 Eicosenoic acid, C18:3: Linolenic acid, C22:0: Behenic acid, C24:0: Lignoceric acid, SFA: Saturated fatty acids, MUFA: Monounsaturated fatty acids, PUFA: Polyunsaturated fatty acids. The asterisk indicates statistically significant differences at a significance level of 5%.

**Table 8:** Eigenvalues and variance explained by the first two principal components for the fatty acids of EVOOs.

Component	Eigenvalue	Difference	Proportion	Cumulative
1	6.317	2.372	0.574	0.574
2	3.945	3.495	0.359	0.933

**Table 9:** Component loadings of the fatty acids of EVOOs.

Variable	Comp1	Comp2	Comp3	Comp4	Comp5	Comp6	Comp7	Unexplained
C14:0	0.395	−0.012	0.053	−0.281	0.169	−0.029	−0.015	0
C16:0	0.295	−0.334	0.017	−0.141	0.154	−0.369	−0.315	0
C17:0	0.370	−0.021	0.358	0.604	−0.470	0.247	−0.179	0
C17:1	0.347	−0.212	0.250	0.108	0.555	0.288	0.326	0
C18:1	−0.119	0.475	0.126	0.058	0.249	0.418	−0.018	0
C18:2	0.275	−0.360	−0.055	−0.052	−0.328	−0.055	0.253	0
C20:1	0.158	0.449	−0.206	0.283	−0.088	−0.518	0.473	0
C20:0	0.159	0.413	0.568	−0.346	−0.108	−0.269	−0.310	0
C18:3	0.339	0.189	−0.473	0.337	0.315	−0.069	−0.497	0
C22:0	0.338	0.194	−0.435	−0.437	−0.351	0.443	−0.021	0
C24:0	0.362	0.203	0.095	−0.120	0.085	−0.023	0.362	0

**Table 10:** Volatile composition (mean ± SD) of Nocellara del Belice and Cerasuola EVOOs for tests T0 (unmodified malaxation) and T20 (modified malaxation, with the application of the SCADA platform). Data are expressed in ppb.

	Nocellara del Belice				Cerasuola			
	T0		T20		T0		T20	
2,4 hexadienal, (E,E)	258	±19.50	569 *	±55.76	415	±32.78	696 *	±61.94
2-pentenal (E)	48	±2.16	58 *	±2.78	40	±1.52	55 *	±3.19
hexanal	124	±8.80	144	±10.14	158	±10.19	200 *	±12.40
pentenal 2-(E)	32	±1.76	41 *	±2.30	91	±5.55	95	±4.56
1-penten-3-ol	48	±1.34	81 *	±1.70	38	±1.026	61 *	±1.10
2-hexen-1-ol	4585	±339.29	6145 *	±380.99	5214	±430.16	9541 *	±776.64
1-hexanol	381	±13.72	414 *	±12.42	535	±12.84	614 *	±13.51
3-hexen-1-ol (Z)	1758	±96.69	1351 *	±63.50	2841	±68.18	2910	±72.75

SD: Standard deviation of three replicates. The asterisk indicates statistically significant differences at a significance level of 5%.

**Table 11:** Eigenvalues and variance explained by the first two principal components for the volatile compounds of EVOOs.

Component	Eigenvalue	Difference	Proportion	Cumulative
1	4.132	2.358	0.689	0.689
2	1.774	1.687	0.296	0.984

**Table 12:** Component loadings of the volatile compounds of EVOOs.

Variable	Comp1	Comp2	Comp3	Comp4	Comp5	Comp6	Unexplained
2,4 hexadienal, (E,E)	0.360	0.495	−0.590	−0.166	0.453	−0.210	0
pentenal 2-(E)	0.013	0.745	0.415	0.030	−0.030	0.520	0
hexanal	0.474	0.183	0.354	0.460	−0.223	−0.596	0
pentenal 2-(E)	0.469	−0.204	−0.385	0.495	−0.218	0.546	0
1-hexanol	0.490	−0.058	0.053	−0.711	−0.497	0.041	0
3-hexen-1-ol (Z)	0.430	−0.349	0.450	−0.100	0.670	0.175	0
